# *Methylomonadaceae* was the active and dominant methanotroph in Tibet lake sediments

**DOI:** 10.1093/ismeco/ycae032

**Published:** 2024-03-04

**Authors:** Yongcui Deng, Chulin Liang, Xiaomeng Zhu, Xinshu Zhu, Lei Chen, Hongan Pan, Fan Xun, Ye Tao, Peng Xing

**Affiliations:** School of Geography, Nanjing Normal University, Nanjing 210023, Jiangsu, China; School of Geography, Nanjing Normal University, Nanjing 210023, Jiangsu, China; School of Geography, Nanjing Normal University, Nanjing 210023, Jiangsu, China; School of Geography, Nanjing Normal University, Nanjing 210023, Jiangsu, China; School of Geography, Nanjing Normal University, Nanjing 210023, Jiangsu, China; School of Geography, Nanjing Normal University, Nanjing 210023, Jiangsu, China; State Key Laboratory of Lake Science and Environment, Nanjing Institute of Geography and Limnology, Chinese Academy of Sciences, Nanjing 210008, Jiangsu, China; University of Chinese Academy of Sciences, Beijing 100039, China; State Key Laboratory of Lake Science and Environment, Nanjing Institute of Geography and Limnology, Chinese Academy of Sciences, Nanjing 210008, Jiangsu, China; State Key Laboratory of Lake Science and Environment, Nanjing Institute of Geography and Limnology, Chinese Academy of Sciences, Nanjing 210008, Jiangsu, China

**Keywords:** Tibetan Plateau, lake sediment, methanotrophs, DNA-SIP, metagenome

## Abstract

Methane (CH_4_), an important greenhouse gas, significantly impacts the local and global climate. Our study focused on the composition and activity of methanotrophs residing in the lakes on the Tibetan Plateau, a hotspot for climate change research. Based on the field survey, the family *Methylomonadaceae* had a much higher relative abundance in freshwater lakes than in brackish and saline lakes, accounting for ~92% of total aerobic methanotrophs. Using the microcosm sediment incubation with ^13^CH_4_ followed by high throughput sequencing and metagenomic analysis, we further demonstrated that the family *Methylomonadaceae* was actively oxidizing CH_4_. Moreover, various methylotrophs, such as the genera *Methylotenera* and *Methylophilus*, were detected in the ^13^C-labeled DNAs, which suggested their participation in CH_4_-carbon sequential assimilation. The presence of CH_4_ metabolism, such as the tetrahydromethanopterin and the ribulose monophosphate pathways, was identified in the metagenome-assembled genomes of the family *Methylomonadaceae*. Furthermore, they had the potential to adapt to oxygen-deficient conditions and utilize multiple electron acceptors, such as metal oxides (Fe^3+^), nitrate, and nitrite, for survival in the Tibet lakes. Our findings highlighted the predominance of *Methylomonadaceae* and the associated microbes as active CH_4_ consumers, potentially regulating the CH_4_ emissions in the Tibet freshwater lakes. These insights contributed to understanding the plateau carbon cycle and emphasized the significance of methanotrophs in mitigating climate change.

## Introduction

Methane (CH_4_) is the second most abundant greenhouse gas in the atmosphere [1], and its concentration has increased from 1.6 ppm in 1983 to 1.9 ppm in 2023 [2]. Inland water ecosystems, including lakes, are significant in CH_4_ emissions, contributing to 6%–16% of total natural CH_4_ emissions [3]. Recent estimations suggest that the annual average release of CH_4_ from lakes accounted for ~18.6% of the global average annual CH_4_ emissions [4].

The Tibetan Plateau, commonly referred to as the “Third Pole” and the “Asian Water Tower,” is highly vulnerable to global warming due to its high elevation. More than half of its area exceeds 4000 m above sea level [5]. This region is home to thousands of lakes, which cover a total area of ~50 323 km^2^ [6], which is roughly 57.2% of China’s lake area [7]. These lakes on the Tibetan Plateau have unique characteristics, such as high altitude, low annual mean temperature [8], and a range of water salinity from freshwater to hypersaline [9]. In a recent study, *in situ* diffusive measurements of CH_4_ flux at the water–air interface of Tibet lakes were conducted [10]. It was found that the diffusive CH_4_ flux in freshwater lakes was 45.14 ± 58.86 μmol·m^−2^·s^−1^, which was 15 times higher than that observed in brackish lakes [10]. These measurements provide valuable insights into the CH_4_ emissions from lakes on the Tibetan Plateau.

CH_4_ emissions in lake sediments are influenced by CH_4_ production and oxidation processes. The major CH_4_ consumers in lakes are aerobic methanotrophs, which can utilize CH_4_ as the sole carbon and energy source [11]. They are capable of consuming up to ∼93% of the CH_4_ produced in the deeper sediments [12]. Aerobic methanotrophs in the phylum *Proteobacteria* can be classified into two types: Type I and Type II [13]. Type I methanotrophs belong to the class *Gammaproteobacteria* and are further categorized into the families *Methylomonadaceae* (*Methylococcaceae*) and *Methylothermaceae*. Type II methanotrophs are members of the class *Alphaproteobacteria* and are mainly affiliated with the families *Methylocystaceae* and *Beijerinckiaceae* [14]. Methanotrophs can also be found in the phylum *Verrucomicrobia* [15]. Oswald et al. suggested that *Crenothrix* could be a relevant CH_4_ consumer in stratified lake water [16]. The particulate methane monooxygenase (pMMO) enzyme, which initiates the first step of CH_4_ oxidation [17], exists in most methanotrophs, with the exception of genera *Methylocella* and *Methyloferula*. The *pmoA* gene, which encodes the alpha subunit of pMMO, is a widely used functional gene to detect methanotrophs [13].

The activity and distribution of methanotrophs in lakes have received significant attention due to their important role in CH_4_ consumption [13, 18]. However, little is known about the proportion of methanotrophs within the bacterial community in the lakes on the Tibetan Plateau. Therefore, the objective of this study is to assess the relative abundance of methanotrophs in Tibet lake sediments and investigate their distribution patterns based on the large-scale sediment sampling across the Tibetan Plateau. In our previous study, methanotroph communities in Tibet lake sediments were dominated by Type I methanotrophs in freshwater lakes, specifically *Methylobacter* and uncultivated Type Ib methanotrophs, while *Methylomicrobium* was prevalent in saline lakes [19]. Salinity was found to be a key factor influencing the composition of aerobic methanotroph communities [19]. However, it remains unclear whether these methanotrophs are actively oxidizing CH_4_ in these sediment environments. To address this question, we used the DNA stable-isotope probing (DNA-SIP) method, successfully identifying metabolically active microorganisms in previous studies [20–22]. Our objective is to determine if the relatively abundant methanotrophs in Tibet lake sediments are actively involved in CH_4_ oxidation.

## Materials and methods

### Lake sediment sampling

**Figure 1:**
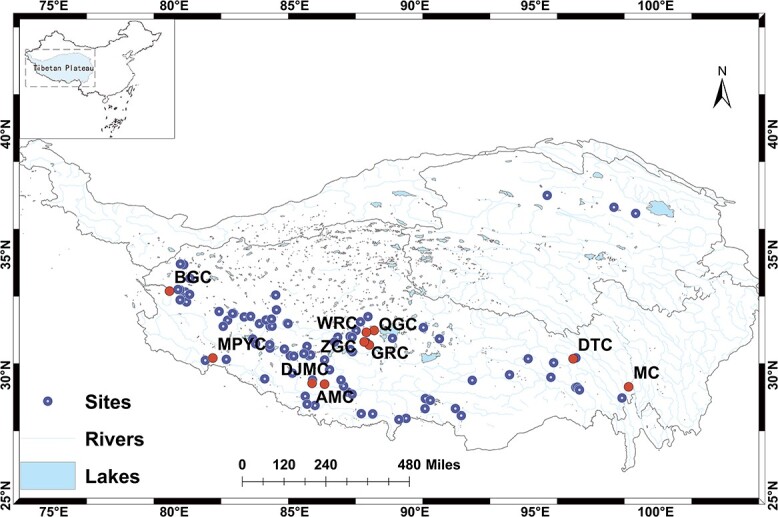
Geographical distribution of the 98 surveyed lakes across the Tibetan Plateau; solid dots represent 10 freshwater lakes with a notably high relative abundance of *Methylomonadaceae* used for DNA-SIP labeling, while hollow dots indicate other sampled lakes within the Tibetan Plateau.

From 2015 to 2020, a total of 231 surface sediment samples were collected from 98 lakes located on the Tibetan Plateau. The geographical distribution of these lakes is shown in Fig. 1. Sediment samples were collected near the maximum water depth using sediment grab samplers. The salinity of the lake water was measured using the portable multiparameter water quality meter (YSI ProQuatro). The 98 investigated lakes were categorized into three groups according to their salinity: freshwater lakes (salinity <0.1%), brackish lakes (0.1% < salinity <3.5%), and saline lakes (salinity >3.5%) [23]. Sediment samples were kept in a cool box during transportation and subsequently stored at a temperature of −20°C in the laboratory for DNA extraction.

### DNA stable-isotope probing incubation

Sediments from 10 lakes, including AmuCo (AMC), BangongCo (BGC), DajiamangCo (DJMC), DatuoCo (DTC), GerenCo (GRC), MangCo (MC), MapangyongCo (MPYC), QigeCo (QGC), WuruCo (WRC), and ZiguiCo (ZGC) with a high relative abundance of *Methylomonadaceae* were incubated in the laboratory. Specifically, 10 g of fresh sediments were added to a 120 ml serum bottle. The bottles were sealed with butyl stoppers after air flushed the headspace. Each lake sediment sample was incubated with >99.99% labeled ^13^CH_4_ or ^12^CH_4_ (as a control), making up 5% of the vial headspace. The incubations were performed in the dark at 15°C for 21 days. CH_4_ concentration was measured daily by gas chromatography (Agilent 7890B). After 7 and 14 days of incubation, headspace gas was refreshed, and CH_4_ was again adjusted to ~5%. The bottles incubated with ^12^CH_4_ were destructively sampled on the 0, 7th, and 21st days of incubation. The bottles incubated with ^13^CH_4_ were sampled only on the seventh day. Two non-sample bottles were set to test the gas tightness of the bottles. After incubation, all subsamples were stored at −20°C for molecular analyses. The following DNA extraction, Quantitative Polymerase Chain Reaction (qPCR), DNA-SIP fractionation, PCR amplification, and high-throughput sequencing methods are detailed in Supplementary 1.

### Sequence analysis of bacterial 16S rRNA and *pmoA* genes

The processing of 16S rRNA data was mainly carried out on the Usearch (https://drive5.com/usearch/) and Mothur (https://mothur.org/) platforms. The paired-end reads were merged first, followed by removing the forward and reverse primers on Usearch, cutting off the 8-bp barcodes using Python. Next, performing quality control, eliminating error sequences greater than 1.0 and leaving unique sequences. The operational taxonomic units (OTUs) were then selected based on 97% sequence similarity, and a OTUs table was created; denoising was necessary for the process. Finally, the taxonomy classification was carried out in Mothur using “classify.seqs.” According to the RDP-v18 reference database, the high-quality sequences were classified based on Wang’s method (cutoff = 80%).

The *pmoA* gene sequencing data were analyzed as previously described [24]. The paired-end sequences were first merged, and the sequence quality was checked in Usearch [25]. Unique sequences were selected from all sequences, and based on 90% similarity, the candidate OTU sequences of the *pmoA* gene were obtained in Usearch. These candidate OTU sequences were imported into ARB (http://www.arb-home.de) [26] to remove the error sequences that cannot be correctly translated into the amino acid sequence. Distance matrices were calculated in ARB based on the 156 amino acid residues of the high-quality *pmoA* sequence. The new OTUs were assigned using a 7% amino acid dissimilarity cutoff using the average linkage algorithm implemented in Mothur. A neighbor-joining phylogenetic tree was constructed in ARB, including the new representative OTU sequences and related reference sequences. Methanotrophic composition data were used to generate a heatmap using the R functions heatmap.2 (R package gplots) [27]. Finally, the phylogenetic tree and heatmap were combined in Adobe Illustrator CS6.

### Co-occurrence network analysis

To investigate the potential interaction of active CH_4_-associated bacterial communities in the labeled DNA of sediment, we conducted the co-occurrence network analysis on the labeled 16S rRNA OTU in ^13^C-DNA (“heavy” fraction). The sixth and seventh fractions of DNAs from ^13^CH_4_ incubation were combined to have sufficient data for the network analysis. The top 100 abundant OTUs were selected for network construction, and the Spearman correlations between each OTU were calculated. Only significant correlations (0.7 < correlation coefficient (ρ) < 0.9, *P*-value <.01) were used, and the networks were created using the “igraph” package in R. For visualization, Gephi software (version 0.9.2; https://gephi.org/) was used, and all these network layouts were generated using the force-based algorithm Hu Yifan.

### Metagenomic sequencing and data analysis

#### Procedures of metagenome sequencing

Three samples, two heavy fractions of DNA of AMC sediment, and the total DNA of MPYC were used for the metagenomic sequencing. At least 1 μg DNA was used for metagenomic library construction following the manufacturer’s instructions of Truseq DNA Library Prep Kits (Illumina, USA). The purified genomic DNA is sheared into smaller fragments with ~400 bp size by Covaris, and blunt ends are generated using T4 DNA polymerase. After adding an “A” base to the 3′ end of the blunt phosphorylated DNA fragments, adapters are ligated to the ends of the DNA fragments. The desired fragments were purified through gel-electrophoresis, then selectively enriched and amplified by PCR. The index tag could be introduced into the adapter at the PCR stage, followed by a library quality test. Finally, the qualified pair-end library would be used for NovaSeq 6000 sequencing (Illumina; 150 bp × 2, Shanghai BIOZERON Co., Ltd). Approximately 20 Gb of raw sequence data were generated for each sample.

#### Metagenome-assembled genome reconstruction, key genes involved in CH_4_ oxidation, nitrogen metabolism, and extracellular electron transfer

First, raw reads for each sample were trimmed using the JAVA program Trimmomatic (version 0.33, http://www.usadellab.org/cms/?page=trimmomatic) to remove sequencing adapters and low-quality sequences (default parameters). Then, MEGAHIT (v.1.1.1, https://github.com/voutcn/megahit; parameter options: --min-contig-len 500 --k-min 21 --k-max 141) was introduced to perform metagenome assembly for each sample. Sequencing depth for each contig (minimum contig length ≥ 1500 bp) was calculated using the functional script “jgi_summarize_bam_contig_depths” from the MetaBAT2 suite (https://bitbucket.org/berkeleylab/metabat) based on the sorted bam files generated by BWA-MEM (v.0.7.17) and SAMtools (v1.546). MetaBAT2 (v.2.12.1), CONCOCT (v0.4.0), and MaxBin (v2.2.4) were applied to bin the assemblies with contig depth results under default parameters. Bins generated by the above three methods were considered the input for the DAS Tool (v1.1.5, https://github.com/cmks/DAS_Tool) to obtain high-quality recovered metagenome-assembled genomes (MAGs). CheckM (v.1.0.7) with lineage_wf workflow was used to estimate the quality of MAGs (completeness and contamination).

The average nucleotide identity (ANI) between each pair of bins was calculated using the OAT JavaScript (http://www.ezbiocloud.net/sw/oat) for all bins. The set of medium-high quality bins obtained from each sample was dereplicated using dRep (https://github.com/ MrOlm/drep, ANI > 95%), resulting in 40 representative genome operational taxonomic units (bins with the highest dRep score, a metric that considers genome sizes, levels of completeness and contamination, strain-heterogeneity, N50 as well as how similar each genome is to all other genomes in their cluster, Supplementary 2). GTDB Toolkit (Genome Taxonomy Database, downloaded Sep 2022, version r207v2) was introduced to obtain the taxonomy information for each MAGs. All of the genes in a bin were transformed to protein sequences to generate the proteomes for each bin to reconstruct the phylogenetic tree using PhyloPhlAN (Supplementary 3) [28].

Prodigal and BLASTP predicted ORFs within MAGs against functional gene databases, including methane cycling genes databases (MCycDB, https://github.com/qichao1984/MCycDB) and nitrogen cycling genes databases (NCycDB, https://github.com/qichao1984/NCyc). Positive hits were considered to have a genome similarity of more than 70% and coverage of more than 60% of BLASTP results. The Kyoto Encyclopedia of Genes and Genomes (KEGG) database was used via the BLASTP program with an E-value cutoff of 10^−5^ further to estimate the function and metabolic pathway of genes.

### Statistics analysis

IBM SPSS Statistics 22 (SPSS Inc., Cary, NC) was used for data analysis. A nonparametric test (Kruskal–Wallis H) was used to test the significant difference in the relative abundance of *Methylomonadaceae* in total bacteria among freshwater lakes (salinity<0.1%), brackish lakes (0.1% < salinity<3.5%), and saline lakes (salinity>3.5%).

## Results

### Relative abundance of *Methylomonadaceae* in Tibet lake sediments

**Figure 2:**
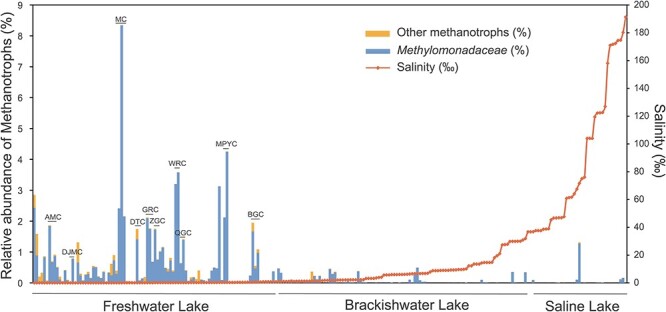
Relative abundance of *Methylomonadaceae* and other methanotrophs categorized by lake salinity; categories include freshwater lakes (salinity < 0.1%), brackish lakes (0.1% < salinity < 3.5%), and saline lakes (salinity > 3.5%); 10 lake samples used for DNA-SIP labeling were listed in the graph.

The relative abundance of *Methylomonadaceae* in lake sediments varied along the salinity gradient (Fig. 2). *Methylomonadaceae* had a significantly higher abundance than other methanotrophs in almost all lakes, accounting for 84.07% of total aerobic methanotrophs (Supplementary 4). The relative abundance of *Methylomonadaceae* in total bacteria ranged from 0% to 8.34%. Freshwater lakes had a significantly higher *Methylomonadaceae* abundance compared to brackish (*P* < .001) and saline lakes (*P* < .001).

In the CH_4_ incubation experiment, we focused on sediment samples from 10 freshwater lakes: MC, MPYC, DJMC, WRC, ZGC, BGC, GRC, DTC, QGC, and AMC, where *Methylomonadaceae* had a relatively higher abundance than other lakes on the Tibetan Plateau (Fig. 2). Among these 10 lakes, the average relative abundance of *Methylomonadaceae* was about 2% in nine lakes, except MC, where *Methylomonadaceae* had the highest relative abundance, reaching 8.34% in bacterial communities.

### CH_4_ oxidation potential and dynamics of *pmoA* genes abundance

**Figure 3:**
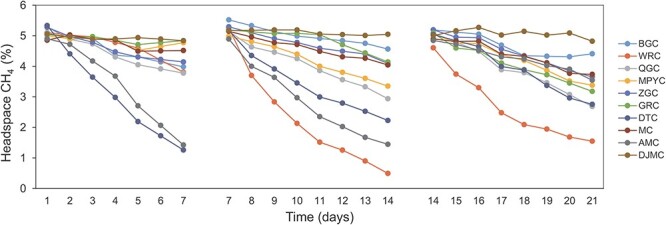
Methane concentrations in bottle headspace during incubation with an initial 5% concertation.

During the 21-day incubation, CH_4_ concentrations decreased to varying extents in all the sediments (Fig. 3). In the first 7 days, the potential for CH_4_ oxidation ranged from 0.216 to 7.78 ng CH_4_ g^−1^ dry weight sediment (d.w.s) day^−1^. The CH_4_ concentration in DTC and AMC bottles decreased dramatically (from ~5% to ~1%), with the methane oxidation rates (MORs) 7.78 and 4.44 ng CH_4_ g^−1^ d.w.s day^−1^, respectively. Between the 7th and 14th days, DTC and AMC bottles consumed CH_4_ dramatically. However, WRC had the highest MOR. From the 14th to the 21st days, WRC still had the highest MOR, while DJMC had the lowest MOR (0.26 ng CH_4_ g^−1^ d.w.s day^−1^), and other lakes had moderate MORs.

**Figure 4:**
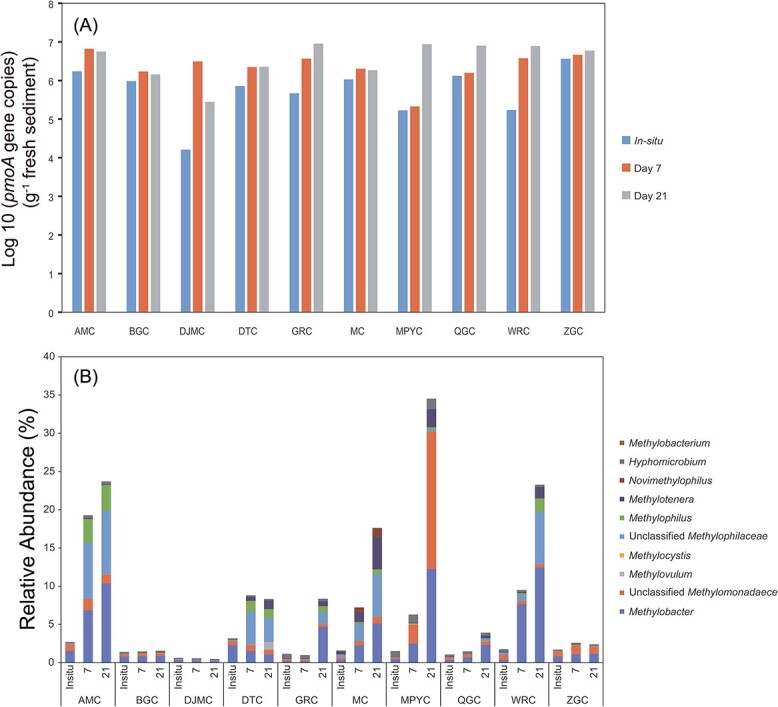
(A) Log 10-converted *pmoA* gene copies in 10 lakes at various incubation times (*in situ*, Day 7, Day 21); (B) relative abundance of methanotrophs based on 16S rRNA gene at these times in the same 10 lakes.

The growth of methanotrophic populations during the CH_4_ incubation was characterized by quantifying the *pmoA* genes using qPCR. The CH_4_ consumption during the incubation was accompanied by the growth of methanotrophic populations, and their abundance varied among lakes (Fig. 4A). On average, the methanotrophs increased from 1.0 × 10^6^ to 5.1 × 10^6^  *pmoA* copies g^−1^ d.w.s after 21 days of incubation. The *pmoA* gene abundance in lake AMC, DJMC, DTC, GRC, MPYC, QGC, and WRC significantly increased after 21-day incubation. Some lakes showed significant increases from *in situ* to Day 7, such as AMC, DJMC, DTC, and WRC. Among them, the *pmoA* gene abundance in DJMC increased the most and reached 1.1 × 10^6^  *pmoA* copies g^−1^ d.w.s. The *pmoA* gene abundance in the other lakes, such as GRC, MPYC, and QGC, increased significantly from Day 7 to Day 21. The *pmoA* gene abundance increased significantly in Lake MPYC, from 2.1 × 10^5^ to 8.7 × 10^6^  *pmoA* copies g^−1^ d.w.s.

### Active methanotrophs in lake sediments

During the sediment incubation, the relative abundance of phylum Proteobacteria increased in lake AMC, DJMC, MC, MPYC, and WRC, especially the classes *Gammaproteobacteria* and *Alphaproteobacteria* (Fig. S1). We further identified the main methanotroph lineages from the total bacteria before and after the CH_4_ incubation (Fig. 4B). Except for lakes BGC and DJMC, the relative abundance of methanotrophs all increased during the incubation. The family *Methylomonadaceae* had the highest relative abundance among the aerobic methanotrophs, with an average proportion of 3.94% in the total bacterial community, even reaching as high as 30.2% in MPYC sediment after 21 days of incubation (Fig. 4B).

**Figure 5:**
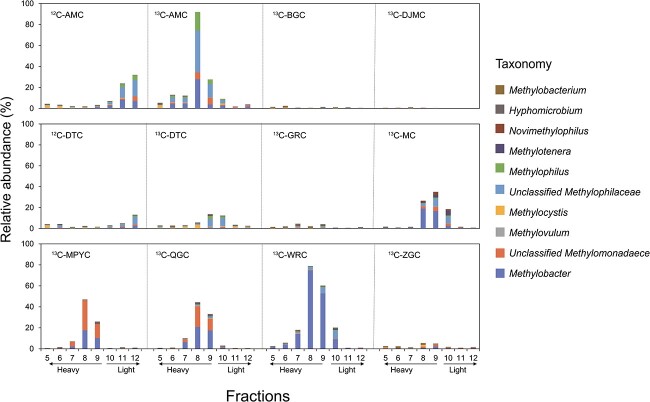
Relative abundance of methylotrophs and methanotrophs based on the 16S rRNA gene in ^12^C- and ^13^C-DNA fractions after a 7-day incubation.

High-throughput sequencing of 16S rRNA revealed the abundance of methanotrophs and other methylotrophs in the heavy fractions of ^13^CH_4_-incubated sediment DNAs (Fig. 5). For example, in AMC lake sediments, the relative abundance of ^13^CH_4_-labeled methanotrophs and other methylotrophs in heavy fraction (Layer 8 in Fig. 5) exceeded 80%. In contrast, the relative abundance of ^12^CH_4_-labeled species was only abundant in the light fraction (Layers 11 and 12 in Fig. 5). Genus *Methylobacter* was the main active methanotroph, and there was also a high percentage of unclassified clusters in *Methylomonadaceae*. Non-methanotrophic methylotrophs, including unclassified clusters in *Methylophilaceae*, *Methylophilus*, and *Methylotenera*, also played an essential role in CH_4_ oxidation. CH_4_-oxidizing methanotrophs in *Methylomonadaceae* were found in 5 out of the 10 tested lakes. The other lakes (BGC, DJMC, DTC, GRC, and ZGC), with some having relatively low CH_4_ oxidation potentials, did not show a high methanotroph abundance in the heavy ^13^C-DNA fractions (Fig. 5).

**Figure 6:**
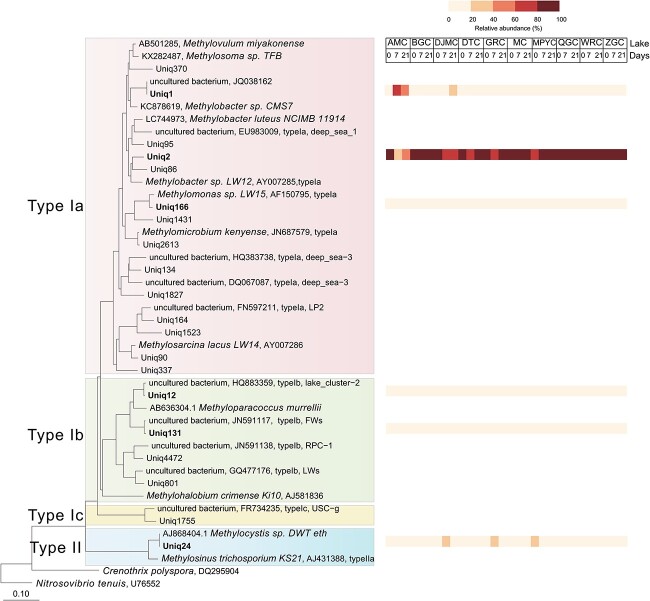
Phylogenetic tree of *pmoA* gene sequences; the tree was constructed based on DNA distance of representative OTUs and related *pmoA* sequences using the Neighbor-joining method on ARB; bold Uniq highlights OTUs with a relative abundance exceeding 1% in lake sediments; a heatmap displays their relative abundance variations across different incubation times, with the transition from low to high relative abundance represented by a color shift from light to dark red.

We also used *pmoA* gene sequencing to classify these methanotrophs further and constructed a DNA-based phylogenetic tree using other *pmoA* genes as references. The *pmoA* sequences utilized in the classification and construction of the phylogenetic tree were sourced from the *pmoA* database [24], as well as NCBI blasting results with 90% identity to the target sequences. This phylogenetic tree included Type Ia, Type Ib, Type Ic, and Type II methanotrophs. Based on 7% amino acid dissimilarity, the *pmoA* OTU representatives were selected, and these OTUs with a relative abundance >1% were included in the phylogenetic tree, including Uniq1, Uniq2, Uniq12, Uniq24, Uniq131, and Uniq166, listed in Fig. 6. Uniq2 was the predominant OTU across nearly all lake sediment samples, with an average relative abundance of 80.9%. The amino acid dissimilarity between Uniq2 and the closest pure culture, *Methylobacter* sp. LW2, measured 7.09%, which was more significant than 7%, suggesting a new species or even genus in methanotrophs (refer to Fig. 2 in Knife, 2015 [13]). Uniq1 dominated the methanotrophs in the 7th and 21st-day incubation AMC samples, closely associating with *Methylobacter* sp. CMS7, exhibiting 6.38% amino acid and 16.56% DNA dissimilarity. Uniq166, proximal to the genus *Methylomonas*, showed a low average relative abundance of 1.11%. Within Type Ib methanotrophs, Uniq12 was found to be in the lake-cluster2, and Uniq131 exhibited proximity to the FWs cluster, both of which had relative abundances below 3.50%. Meanwhile, Uniq 24 was affiliated with the *Methylocystis* genus, exhibiting an average relative abundance of 5.47%. It was prominently observed in the 7-day incubated DJMC sample, 7-day incubated GRC sample, and 21-day incubated MPYC sample.

### Co-occurrence network of methanotrophs and other bacteria

**Figure 7:**
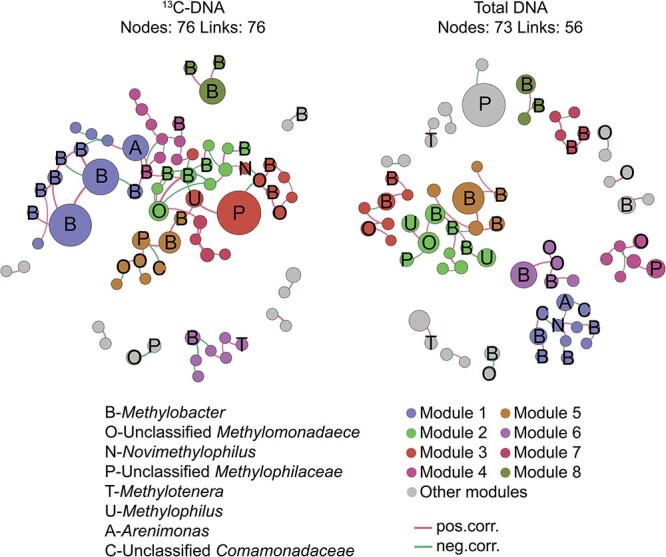
Bacterial co-occurrence network in ^13^C-CH_4_-labeled DNA and total DNA of CH_4_-incubated lake sediments; abundant OTUs with > 0.15% average relative abundance were selected to construct the networks, with red and green lines indicating positive and negative correlations, respectively; node size reflects the “degree” (number of connections), and the top eight modules were displayed.

Co-occurrence networks based on the abundant 16S rRNA gene OTUs from the ^13^C-DNA heavy fractions and total DNA were constructed, respectively (Fig. 7). In the ^13^C-DNA network (Fig. 7), 29 OTUs were methanotrophs, and 6 OTUs were methylotrophs among the 76 nodes. Significant correlations among OTUs affiliated with B-*Methylobacter* OTUs were detected in Modules 1, 2, and 4. Besides, OTUs of unclassified P-*Methylophilaceae* enriched in Modules 3 and 5 were positively correlated with B-*Methylobacter* OTUs and unclassified O-*Methylomonadaece* OTUs. Besides the correlation between methanotrophs and methylotrophs, the correlation between methanotrophs and heterotrophs reveals a complex metabolic correlation in the ^13^C-DNA network. For example, A-*Arenimonas* OTUs, as a denitrifying bacterium, were positively correlated with B-*Methylobacter* OTUs in Module 1.

In the total DNA network (Fig. 7), 36 OTUs and 8 OTUs were methanotrophs and other methylotrophs, respectively. In Module 1, significant correlations between methylotrophs (N-*Novimethylophilus* and B-*Methylobacter* OTUs) and denitrifying bacteria (unclassified C-*Comamonadaceae* and A-*Arenimonas* OTUs) were detected. Module 2 showed the positive correlation between OTUs B-*Methylobacter*, unclassified O-*Methylomonadaece*, and U-*Methylophilus*. Significant positive correlations among B-*Methylobacter* OTUs and unclassified O-*Methylomonadaece* were detected in Module 4.

### *Methylomonadaceae metagenome-assembled genome* and its metabolic adaption

Five representatives MAGs were identified as belonging to methanotrophs, and three belonged to methylotrophs (Supplementary 2). To construct a phylogenetic tree of the family *Methylomonadaceae*, the MAGs (Bin_009, Bin_018, Bin_025, Bin_038, and Bin_041) were compared with reference MAGs downloaded from NCBI (Supplementary 3). The tree included various genera such as *Methylomonas*, *Methylobacter*, *Methylocaldum*, *Methylococcus*, *Methylovulum*, *Methylomicrobium*, and KS41. It is worth noting that Bin_025 was the most abundant MAGs in AMC samples, and it could not be classified at the genus level (belonging to uncultured cluster_*Methylomonadaece*_JABFRC01, Supplementary 3). Bin_009 affiliated with the genus *Methylovulum* and showed a close relationship with *Methylovulum oryzae*. Bin_041, the second most abundant MAG in AMC, belonged to the family *Methylomonadaceae* and the genus *Methylobacter*. Bin_041 contained the *porB/D* gene (Serine cycle) in the CH_4_ metabolic module and the *mvhA* gene (Central methanogenic pathway) in the CH_4_ metabolic module, which were not found in other genomes in the genus *Methylobacter*. Bin_018 and Bin_038 were also identified in the AMC and MPYC sediment; both belonged to the family *Methylomonadaceae* at the genus level KS41.

**Figure 8:**
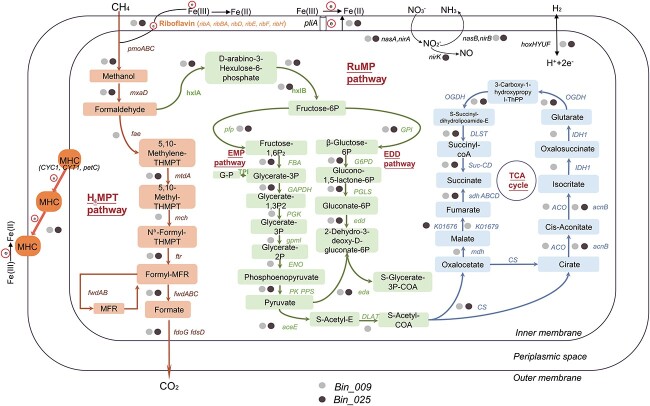
Metabolic pathway of methanotrophs. Bin_009 and Bin_025 in the family *Methylomonadaceae*, annotated with completeness of 99.6% and 73.7%, respectively, were selected to construct the CH_4_ metabolic pathway; gray and black dots represent gene detection; the absence of dots indicates no detection.

To further study the metabolism of *Methylomonadaece*, we analyzed the metabolic pathways of two MAGs (Bin_025 and Bin_009), which were enriched in AMC and MPYC, respectively (Supplementary 2, Fig. 8). Bin_025 was annotated as *Methylomonadaece*_JABFRC01, and Bin_009 was annotated as *Methylomonadaece*_*Methylovolum*. Regarding CH_4_ oxidation, both Bin_025 and Bin_009 contained gene clusters (*pmoCAB*) encoding pMMO. No genes encoding the soluble methane monooxygenase (sMMO, *mmoXYBZDC*) were detected. In the process of methanol dehydrogenation to formaldehyde, these two MAGs only contain calcium-dependent (*mxaFJGID*) dehydrogenases and do not have enzymes with *XoxF* lanthanide. In the tetrahydromethanopterin (H_4_MPT) pathway that converts formaldehyde to formate, genes encoding tetrahydromethanopterin hydrolase (*fae*), methylenetetrahydrofolate, methenyltetrahydromethanopterin cyclohydrolase (*mch*), formylmethanofuran (*ftr*), and formylmethanofuran dehydrogenase (*fwdAB*) were present completely. In terms of formaldehyde assimilation pathways, Bin_009 contained three pathways: the ribulose monophosphate (RuMP), the Embden–Meyerhof–Parnas (EMP), and the Enter–Doudoroff (ED) pathways. However, Bin_025 lacked essential genes (PGK, gpml, ENO), resulting in an incomplete EMP pathway.

Regarding nitrogen metabolism, Bin_025 and Bin_009 possess genes involved in denitrification and assimilation (Fig. 8). The process of nitrate conversion to nitrite (NO_3_^−^ → NO_2_^−^) can be accomplished by various enzymes, including assimilatory nitrate reductase (NAS, *nasA,* and *nirA*), respiratory nitrate reductase (NAR, *narGH*), periplasmic nitrate reductases (NAP, *napA*), as well as nitrite oxidoreductase (NXR, *nxrAB*). Only genes encoding NAS were found in Bin_009 and Bin_025, while genes encoding NXR were detected in Bin_025. Regarding the process of nitrite conversion to nitric oxide (NO_2_^−^ → NO), there are two categories of nitrite reductase (NIR): a copper-containing NIR (Cu-NIR) encoded by *nirK* gene and a cytochrome *cd_1_*-containing NIR (*cd_1_*-NIR) encoded by *nirS* gene. The *nirK* gene encoding copper-containing (Cu-NIR) nitrite reductases was detected in Bin_025. Genes encoding the assimilatory NIR (cNIR; *nasB* and *nirB*) involved in reducing nitrite to NH_3_ (NO_2_^−^ → NH_3_) were present in our four MAGs, except for Bin_018.

Regarding hydrogen metabolism, Bin_025 and Bin_009 contain the genes *hoxH*, *hoxY*, *hoxU*, and *hoxF*, which are involved in bidirectional hydrogenase (Fig. 8). Additionally, cyc1, the ubiquinol-cytochrome c reductase cytochrome c1 subunit, suggests a potential involvement in iron oxidation. These five MAGs contain genes involved in extracellular electron transfer (EET). EET is a process by which microorganisms exchange electrons with their environment, enabling them to transfer energy and perform various metabolic activities. This process involves three main pathways: multiheme c type cytochromes (MHCs), nanowires, and electron shuttles [29]. First, the *pilA* gene encoding electrically conductive pili (e-pili) was detected in Bin_025 and Bin_009. Second, the genes encoding MHCs (*CYC1*, *CYT1*, *petC*) were also identified in Bin_025 and Bin_009. Lastly, the genes encoding riboflavin (*ribA*, *ribBA*, *ribD*, *ribE*, *ribF*, and *ribH*), which serve a typical electron shuttle, were detected in all five MAGs.

## Discussion

### Salinity had a significant effect on the CH_4_ cycle in Tibet lakes

In the studied Tibet lakes, a much higher relative abundance of *Methylomonadaceae* was detected in the freshwater lakes compared to brackish and saline lakes (Fig. 2). Significant effects of salinity on methanotroph community composition in Tibet lakes and other lake sediments were reported previously [19]. *Methylomicrobium* and other Type Ia salt-tolerant and halophilic methanotrophs play an active role in CH_4_ cycling [19, 30–32]. For example, *Methylomicrobium* became dominant with the increase in salinity, and the previously inhibited methanotrophy quickly recovered in Lake Qinghai sediment [33]. In this study, we found even though methanotrophs like *Methylomicrobium* exist widely and were actively oxidizing CH_4_ in saline lakes, their relative abundance in bacteria was generally low (<0.1%). Like methanotrophs, the relative abundance of methanogens in Tibet lake sediments was also lower in brackish water and saline lakes than in freshwater lakes [34]. These findings suggest that CH_4_-cycling microorganisms, including methanogens and methanotrophs, are less abundant in brackish water and saline lakes than in freshwater lakes.

Lakes were estimated to contribute ~18.6% of the global average annual CH_4_ emissions [4]. *In situ* measurements of lake CH_4_ flux in the Tibet lakes have shown that CH_4_ emissions are much higher in freshwater lakes than in brackish and saline lakes [10]. This suggests that the relative abundance and the activity of methanotrophs and methanogens were reduced according to salinization. Therefore, considering the CH_4_ flux and the relative abundance of methanotrophs and methanogens, the contribution of freshwater lakes to CH_4_ cycling is higher compared to that of brackish and saline lakes.

Global warming, increased precipitation, and accelerated melting of glaciers and permafrost have led to an expansion of more than 80% of the lake area in the Qinghai-Tibet Plateau. For example, from 1979 to 2017, Lake Selincuo increased from 1667 km^2^ to 2389 km^2^ [35]. The expansion of lakes has diluted their salinity, which seems beneficial for the survival of methanogens and methanotrophs and has the potential to stimulate CH_4_ production and oxidation, thereby increasing the contribution of these brackish water and saline lakes to CH_4_ cycling.

### *Methylomonadaceae* is the active CH_4_ oxidizer in Tibet lake

In the previous study, *Methylomonadaceae* was identified as an abundant family in lake sediments on the Tibetan Plateau [19]. Using DNA-SIP, 16S rRNA amplified sequencing, and metagenome analysis, we found the potentially new genus within *Methylomonadaceae* that was not only abundant but also actively involved in CH_4_ oxidation in these cold, high-altitude lake sediments (Figs 2, 5, and 6). This was supported by detecting abundant unique sequences Uniq1 and Uniq2 from pmoA sequencing and identifying potentially new genus Bin_025 through metagenomic binning analysis. Previous research has also explored active methanotrophs in lake sediments using DNA-SIP in various locations worldwide. These include lakes in China, England [36], India [31], Russia [37], Germany [38], and North America [21, 22, 39]. *Methylomonadaece* is the dominant group responsible for CH_4_ oxidation in all these studies. For example, in German lake sediments, Dumont et al. identified *Methylobacter* as the most active methanotroph, a genus within the family *Methylomonadaece* [38]. In China and England, Yang et al. found that *Crenothrix*, also belonging to the family *Methylomonadaece*, was the dominant methanotroph in two lake sediments [36]. In North America, He et al. identified *Methylomonas*, *Methylobacter*, and *Methylosoma*, all within the family *Methylomonadaece*, as active methanotrophs in lakes [21, 22, 39]. In saline water lakes, abundant microorganisms, including *Methylomicrobium* and *Methylobacter*, which also belong to the family *Methylomonadaece*, are dominant [31, 40].

Our DNA-SIP experiments found that certain MAGs within the family *Methylomonadaece* contained a complete CH_4_ metabolic pathway, including the H_4_MPT pathway, RuMP pathway, and the tricarboxylic acid cycle. Methanotrophs exhibit remarkable metabolic flexibility and can adapt to oxygen-deprived conditions. In anaerobic conditions, anaerobic methanotrophs can utilize various electron acceptors, such as sulfate, metal oxides like iron (Fe^3+^) and manganese (Mn^4+^), nitrate, nitrite, and arsenate, for the process of coupled CH_4_ oxidation [40–43]. In the meantime, in O_2_-limited conditions, aerobic methanotrophs from the gammaproteobacterial group dominated the methanotrophic community and exhibited activity in freshwater lakes. Their CH_4_ oxidation was also stimulated by adding iron and manganese oxides [16]. Under O_2_-limited conditions, iron oxides can be an alternative electron acceptor for methanotrophs [39, 44–46]. However, since cells cannot take up solid iron oxides, EET is essential in microbial iron reduction. Genes encoding EET mentioned above were all detected in Bin_025 and Bin_009 (Fig. 8). Riboflavin is a typical kind of electron shuttle [47]. With the help of riboflavin, the enriched methanotrophs consortium used ferric oxides as alternative electron acceptors for oxidizing CH_4_ when O_2_ was unavailable [45, 47].

Molecular hydrogen is considered alternative energy conservation in the energetic input of H_2_, which might counter the effect of otherwise unbalanced growth conditions, such as the O_2_-limited environment [48]. Lake sediments are O_2_-limited environments, and from the MAG results, it appears that methanotrophs have genes *hoxHYUF* that are potentially involved in bidirectional hydrogenase.

Methanotrophs with nitrogen metabolism genes may utilize NO_3_^−^ as an alternative electron acceptor when O_2_ is limited. It is worth mentioning that no aerobic methanotrophs that have been isolated in pure culture have been demonstrated to perform the function of complete denitrification (NO_3_^−^ → NO_2_^−^ → NO→N_2_O → N_2_) [49]. However, an obligate aerobic methanotrophic bacterium, *Methylomonas denitrificans* FJG1, has the genes encoding the nitrate reductase NAR and has been demonstrated to couple partial denitrification with CH_4_ oxidation, producing nitrous oxide as a terminal product under hypoxia conditions [49]. In our study, the genes encoding the reductases involved in the first (NAS, *nasA*, and *nirA*) and second (Cu-NIR, *nirK*) steps of denitrification (NO_3_^−^ → NO_2_^−^ → NO) were detected in *Methylomonadaece* MAGs of Tibet lakes, suggesting their potential to conduct the denitrification. Therefore, methanotrophs influence global change by impacting carbon and nitrogen cycling in ecosystems.

### Cross-feeding among the methanotrophs and the associated bacteria

Our study revealed that lake sediments contained methylotrophs, including *Methylotenera*, *Methylophilus*, and unclassified clusters in the *Methylophilaceae* family, as detected in the ^13^C-labeled DNAs (Fig. 7). These methylotrophs utilize methanol, an intermediate CH_4_ oxidation metabolite, as a carbon source [22]. This finding is consistent with previous research that observed an abundance of methylotrophs in the ^13^C-labeled DNA due to their involvement in CH_4_-carbon assimilation [21, 22, 50–52].

Except for methylotrophs, other types of metabolic interactions were also identified and shown in the ^13^C-labeled DNA network. This includes interactions between denitrification and CH_4_ oxidation. In aerobic conditions, CH_4_ oxidation coupled to denitrification can be described as aerobic CH_4_ oxidation coupled to denitrification (AME-D). Denitrifying bacteria is essential in the process of reducing nitrate to nitrite. They could utilize metabolites such as formaldehyde, formate, and particularly methanol, produced by methanotrophs as substrates in aerobic CH_4_ oxidation coupled with denitrification [53–55]. During the ^13^CH_4_ incubation, taxa in the genus *Arenimonas* and family *Comamonadaceae* were labeled (Fig. 7). These heterotrophic denitrifiers have been observed to engage in syntrophy with aerobic methanotrophs (primarily *Methylobacter*, Fig. 7) under aerobic and microaerobic conditions [56].

## Conclusion

*Methylomonadaceae*, specifically some new clusters close to the genus *Methylobacter*, is the dominant and active CH_4_ oxidizer in the lake sediments on the Tibetan Plateau. The relative abundance of *Methylomonadaceae* is higher in freshwater lakes compared to brackish and saline lakes. Other methylotrophs, such as *Methylotenera* and *Methylophilus*, are also present and play a role in CH_4_ carbon assimilation. There are metabolic interactions between methanotrophs and other bacteria, including denitrifying bacteria, indicating a potential coupling of CH_4_ oxidation and denitrification in the sediments. The results suggest that the CH_4_-oxidizing microorganisms in the lake sediments are influenced by salinity and play a significant role in the CH_4_ cycles from these lakes.

## Supplementary Material

Supplementary_1_ycae032

Supplementary_2_ycae032

Supplementary_3_ycae032

Supplementary_4_ycae032

## Data Availability

All the raw sequencing data were submitted to SRA with Project No. PRJNA1048174 and PRJNA1056829. The metagenome raw reads were deposited into the NCBI database with the Accession Numbers SUB14101129 and SUB14105025.

## References

[1] IPCC . Summary for policymakers. In: Masson-Delmotte V, Zhai P, Pirani JA (ed.), Climate Change 2021: The Physical Science Basis. Contribution of Working Group I to the Sixth Assessment Report of the Intergovernmental Panel on Climate Change. Cambridge University Press, Cambridge, United Kingdom and New York, NY, USA, In press. 2021. https://www.ipcc.ch/report/ar6/wg1/

[2] National Oceanic and Atmospheric Administration . The NOAA Annual Greenhouse Gas Index (AGGI). 2023. Retrieved from https://gml.noaa.gov/aggi/aggi.html.

[3] Bastviken D, Tranvik LJ, Downing JA. et al. Freshwater methane emissions offset the continental carbon sink. Scienc*e* 2011;331:50–0. 10.1126/science.1196808 21212349

[4] Rosentreter JA, Borges AV, Deemer BR. et al. Half of global methane emissions come from highly variable aquatic ecosystem sources. Nat Geosc*i* 2021;14:225–30. 10.1038/s41561-021-00715-2

[5] Kuang X, Jiao JJ. Review on climate change on the Tibetan Plateau during the last half century. J Geophys Res Atmo*s* 2016;121:3979–4007. 10.1002/2015JD024728

[6] Yao T, Bolch T, Chen D. et al. The imbalance of the Asian water tower. Nat Rev Earth Enviro*n* 2022;3:618–32. 10.1038/s43017-022-00299-4

[7] Zhang G, Yao T, Xie H. et al. Response of Tibetan Plateau lakes to climate change: trends, patterns, and mechanisms. Earth-Sci Re*v* 2020;208:103269. 10.1016/j.earscirev.2020.103269

[8] Zheng M . An Introduction to Saline Lakes on the Qinghai—Tibet Platea*u*. Springer Dordrecht, Dordrecht, 1997. 10.1007/978-94-011-5458-1

[9] Song K, Wang M, Du J. et al. Spatiotemporal variations of lake surface temperature across the Tibetan Plateau using MODIS LST product. Remote Sen*s* 2016;8:854. 10.3390/rs8100854

[10] Xun F, Li B, Chen H. et al. Effect of salinity in alpine lakes on the southern Tibetan Plateau on greenhouse gas diffusive fluxes. J Geophys Res Biogeosc*i* 2022;127:e2022JG006984. 10.1029/2022jg006984

[11] Borrel G, Jézéquel D, Biderre-Petit C. et al. Production and consumption of methane in freshwater lake ecosystems. Res Microbio*l* 2011;162:832–47. 10.1016/j.resmic.2011.06.004 21704700

[12] Frenzel P, Thebrath B, Conrad R. Oxidation of methane in the oxic surface layer of a deep Lake sediment (lake Constance). FEMS Microbiol Eco*l* 1990;73:149–58. 10.1111/j.1574-6968.1990.tb03935.x

[13] Knief C . Diversity and habitat preferences of cultivated and uncultivated aerobic methanotrophic bacteria evaluated based on *pmoA* as molecular marker. Front Microbio*l* 2015;6:1346. 10.3389/fmicb.2015.01346 26696968 PMC4678205

[14] Belova SE, Oshkin IY, Glagolev MV. et al. Methanotrophic bacteria in cold seeps of the floodplains of northern rivers. Microbiolog*y* 2013;82:743–50. 10.1134/S0026261713060040 25509412

[15] Dunfield PF, Yuryev A, Senin P. et al. Methane oxidation by an extremely acidophilic bacterium of the phylum Verrucomicrobia. Natur*e* 2007;450:879–82. 10.1038/nature06411 18004300

[16] Oswald K, Graf JS, Littmann S. et al. *Crenothrix* are major methane consumers in stratified lakes. ISME *J* 2017;11:2124–40. 10.1038/ismej.2017.77 28585934 PMC5563964

[17] Hakemian AS, Rosenzweig AC. The biochemistry of methane oxidation. Annu Rev Bioche*m* 2007;76:223–41. 10.1146/annurev.biochem.76.061505.175355 17328677

[18] Chistoserdova L, Kalyuzhnaya MG. Current trends in methylotrophy. Trends Microbio*l* 2018;26:703–14. 10.1016/j.tim.2018.01.011 29471983

[19] Deng Y, Liu Y, Dumont M. et al. Salinity affects the composition of the aerobic methanotroph community in alkaline lake sediments from the Tibetan plateau. Microb Eco*l* 2017;73:101–10. 10.1007/s00248-016-0879-5 27878346

[20] Dumont MG, Pommerenke B, Casper P. et al. DNA-, rRNA- and mRNA-based stable isotope probing of aerobic methanotrophs in lake sediment: stable isotope probing of methanotrophs. Environ Microbio*l* 2011;13:1153–67. 10.1111/j.1462-2920.2010.02415.x 21261798

[21] He R, Wooller MJ, Pohlman JW. et al. Shifts in identity and activity of methanotrophs in Arctic lake sediments in response to temperature changes. Appl Environ Microbio*l* 2012;78:4715–23. 10.1128/aem.00853-12 22522690 PMC3370501

[22] He R, Wooller MJ, Pohlman JW. et al. Methane-derived carbon flow through microbial communities in arctic lake sediments. Environ Microbio*l* 2015;17:3233–50. 10.1111/1462-2920.12773 25581131

[23] Song C, Sun L, Huang Y. et al. Carbon exchange in a freshwater marsh in the Sanjiang Plain, northeastern China. Agric For Meteoro*l* 2011;151:1131–8. 10.1016/j.agrformet.2011.04.001

[24] Deng YC, Cui XY, Lüke C. et al. Aerobic methanotroph diversity in Riganqiao peatlands on the Qinghai-Tibetan Plateau. Environ Microbiol Re*p* 2013;5:566–74. 10.1111/1758-2229.12046 23864571

[25] Edgar RC . Search and clustering orders of magnitude faster than BLAST. Bioinformatic*s* 2010;26:2460–1. 10.1093/bioinformatics/btq461 20709691

[26] Ludwig W, Strunk O, Westram R. et al. ARB: a software environment for sequence data. Nucleic Acids Res 2004;32:1363–71. 10.1093/nar/gkh293 14985472 PMC390282

[27] Warnes GR, Bolker B, Bonebakker L. et al. gplots: Various R Programming Tools for Plotting Data. R package version 3.1.3.1. 2024. https://CRAN.R-project.org/package=gplotsWarnes

[28] Segata N, Börnigen D, Morgan XC. et al. PhyloPhlAn is a new method for improved phylogenetic and taxonomic placement of microbes. Nat Commu*n* 2013;4:2304. 10.1038/ncomms3304 23942190 PMC3760377

[29] Lovley DR . Syntrophy goes electric: direct interspecies electron transfer. Ann Rev Microbio*l* 2017;71:643–64. 10.1146/annurev-micro-030117-020420 28697668

[30] Khmelenina VN, Shchukin VN, Reshetnikov AS. et al. Structural and functional features of methanotrophs from hypersaline and alkaline lakes. Microbiolog*y* 2010;79:472–82. 10.1134/S0026261710040090

[31] Antony CP, Kumaresan D, Ferrando L. et al. Active methylotrophs in the sediments of Lonar Lake, a saline and alkaline ecosystem formed by meteor impact. ISME J*.* 2010;4:1470–80. 10.1038/ismej.2010.70 20555363

[32] Shiau Y-J, Lin C-W, Cai Y. et al. Niche differentiation of active methane-oxidizing bacteria in estuarine mangrove forest soils in Taiwan. Microorganism*s* 2020;8:2076–607. 10.3390/microorganisms8081248 PMC7466156 32824517

[33] Fang J, Adams JM, Deng Y. et al. Propagule limitation affects the response of soil methane oxidizer community to increased salinity. Geoderm*a* 2022;426:116082. 10.1016/j.geoderma.2022.116082

[34] Liu Y, Priscu JC, Xiong J. et al. Salinity drives archaeal distribution patterns in high altitude lake sediments on the Tibetan Plateau. FEMS Microbiol Eco*l* 2016;92:fiw033. 10.1093/femsec/fiw033 26887660

[35] Zhang Z, Chang J, Xu C-Y. et al. The response of lake area and vegetation cover variations to climate change over the Qinghai-Tibetan Plateau during the past 30 years. Sci Total Enviro*n* 2018;635:443–51. 10.1016/j.scitotenv.2018.04.113 29677670

[36] Yang Y, Chen J, Pratscher J. et al. DNA-SIP reveals an overlooked methanotroph, *Crenothrix* sp., involved in methane consumption in shallow lake sediments. Sci Total Enviro*n* 2022;814:152742. 10.1016/j.scitotenv.2021.152742 34974014

[37] Lin J-L, Radajewski S, Eshinimaev BT. et al. Molecular diversity of methanotrophs in Transbaikal soda lake sediments and identification of potentially active populations by stable isotope probing. Environ Microbio*l* 2004;6:1049–60. 10.1111/j.1462-2920.2004.00635.x 15344930

[38] Dumont MG, Pommerenke B, Casper P. Using stable isotope probing to obtain a targeted metatranscriptome of aerobic methanotrophs in lake sediment. Environ Microbiol Re*p* 2013;5:757–64. 10.1111/1758-2229.12078 24115627

[39] He R, Wang J, Pohlman JW. et al. Metabolic flexibility of aerobic methanotrophs under anoxic conditions in Arctic lake sediments. ISME *J* 2022;16:78–90. 10.1038/s41396-021-01049-y 34244610 PMC8692461

[40] Su G, Zopfi J, Yao H. et al. Manganese/iron-supported sulfate-dependent anaerobic oxidation of methane by archaea in lake sediments. Limnol Oceanog*r* 2020;65:863–75. 10.1002/lno.11354

[41] Shi L-D, Lv P-L, Mcilroy SJ. et al. Methane-dependent selenate reduction by a bacterial consortium. ISME *J* 2021;15:3683–92. 10.1038/s41396-021-01044-3 34183781 PMC8630058

[42] Ettwig KF, Butler MK, Le Paslier D. et al. Nitrite-driven anaerobic methane oxidation by oxygenic bacteria. Natur*e* 2010;464:543–8. 10.1038/nature08883 20336137

[43] Ettwig KF, Zhu BL, Speth D. et al. Archaea catalyze iron-dependent anaerobic oxidation of methane. Proc Natl Acad Sci U S *A* 2016;113:12792–6. 10.1073/pnas.1609534113 27791118 PMC5111651

[44] Zheng Y, Wang H, Liu Y. et al. Methane-dependent mineral reduction by aerobic methanotrophs under hypoxia. Environ Sci Technol Let*t* 2020;7:606–12. 10.1021/acs.estlett.0c00436

[45] Li B, Tao Y, Mao Z. et al. Iron oxides act as an alternative electron acceptor for aerobic methanotrophs in anoxic lake sediments. Water Re*s* 2023;234:119833. 10.1016/j.watres.2023.119833 36889095

[46] Bar-Or I, Elvert M, Eckert W. et al. Iron-coupled anaerobic oxidation of methane performed by a mixed bacterial-archaeal community based on poorly reactive minerals. Environ Sci Techno*l* 2017;51:12293–301. 10.1021/acs.est.7b03126 28965392

[47] Sepulveda Cisternas I, Salazar JC, Garcia-Angulo VA. Overview on the bacterial iron-riboflavin metabolic axis. Front Microbio*l* 2018;9:1478. 10.3389/fmicb.2018.01478 30026736 PMC6041382

[48] Carere CR, Mcdonald B, Peach HA. et al. Hydrogen oxidation influences glycogen accumulation in a Verrucomicrobial methanotroph. Front Microbio*l* 2019;10:2111–9. 10.3389/fmicb.2019.01873 31474959 PMC6706786

[49] Kits KD, Klotz MG, Stein LY. Methane oxidation coupled to nitrate reduction under hypoxia by the *Gammaproteobacterium Methylomonas denitrificans*, sp. nov. type strain FJG1. Environ Microbio*l* 2015;17:3219–32. 10.1111/1462-2920.12772 25580993

[50] Graef C, Hestnes AG, Svenning MM. et al. The active methanotrophic community in a wetland from the high Arctic. Environ Microbiol Re*p* 2011;3:466–72. 10.1111/j.1758-2229.2010.00237.x 23761309

[51] Jensen S, Neufeld JD, Birkeland N-K. et al. Methane assimilation and trophic interactions with marine *Methylomicrobium* in deep-water coral reef sediment off the coast of Norway. FEMS Microbiol Eco*l* 2008;66:320–30. 10.1111/j.1574-6941.2008.00575.x 18811651

[52] Krause SMB, Johnson T, Samadhi Karunaratne Y. et al. Lanthanide-dependent cross-feeding of methane-derived carbon is linked by microbial community interactions. Proc Natl Acad Sci U S *A* 2017;114:358–63. 10.1073/pnas.1619871114 28028242 PMC5240692

[53] Zhu J, Wang Q, Yuan M. et al. Microbiology and potential applications of aerobic methane oxidation coupled to denitrification (AME-D) process: a review. Water Re*s* 2016;90:203–15. 10.1016/j.watres.2015.12.020 26734780

[54] Rhee GY, Fuhs GW. Wastewater denitrification with one-carbon compounds as energy source. Water Pollution Control Federation 1978;50:2111–9.

[55] Alrashed W, Lee J, Park J. et al. Hypoxic methane oxidation coupled to denitrification in a membrane biofilm. Chem Eng *J* 2018;348:745–53. 10.1016/j.cej.2018.04.202

[56] Guo X, Lai C-Y, Hartmann EM. et al. Heterotrophic denitrification: an overlooked factor that contributes to nitrogen removal in n-DAMO mixed culture. Environ Re*s* 2023;216:114802. 10.1016/j.envres.2022.114802 36375502

