# Supplementary material for: Methylomonadaceae was the active and dominant methanotroph in Tibet lake sediments
Source: ISME Commun. 2024 Mar 4;4(1):ycae032. doi: 10.1093/ismeco/ycae032 (PMC10960969; doi:10.1093/ismeco/ycae032)
Supplement: Supplementary_1_ycae032 [file supplementary_1_ycae032.docx]

Submitted to ISME communications

**Supplementary 1**

***Methylomonadaceae* was the active and dominant methanotroph in Tibet lake sediments**

Yongcui Deng^1^, Chulin Liang^1^, Xiaomeng Zhu^1^, Xinshu Zhu^1^, Lei Chen^1^, Hongan Pan^1^, Fan Xun^2,3^, Ye Tao^2^, Peng Xing^2, *^

^1^ School of Geography, Nanjing Normal University, Nanjing 210023, China

^2^ State Key Laboratory of Lake Science and Environment, Nanjing Institute of Geography and Limnology, Chinese Academy of Sciences, Nanjing 210008, China

^3^ University of Chinese Academy of Sciences, Beijing 100039, China

Running title: *Methylomonadaceae* dominate Tibet lakes

^∗^Corresponding author. No. 73 East Beijing Road, Nanjing 210008, China. E-mail: [pxing@niglas.ac.cn](mailto:pxing@niglas.ac.cn). Fax: 86 25 57714759

**DNA extraction and Quantitative PCR**

After incubation, DNA was extracted from 0.5 g of sediment using the FastDNA SPIN Kit (MP Biomedicals, USA), and the extracted DNA concentration was measured with an Onedrop ultraviolet spectrophotometer. The qPCR was used to detect the abundance of methanotrophs in the sediments during incubation. The *pmoA* gene quantitative PCR (qPCR) was performed using the A189F (Holmes et al. 1995)/mb661r (Costello et al. 1999) primer pairs. Each reaction (20 μL volumes) consisted of 10 μL of 2× SYBR Green Pro Taq HS Premix (Accurate Biotechnology Co., Ltd, China), 0.4 μL of each primer (10 μM), 0.4 μL of ROX Reference Dye (4 μM), and 2 μL of template DNA. The qPCR was carried out on an ABI QauntStudio Real-Time Detection System (Applied Biosystems, USA) using an initial denaturation at 95 °C for 30 s, followed by 40 cycles of denaturation at 95 °C for 5 s, annealing, and extension at 62 °C for 30 s. The melting curve analysis (95 °C, 15 s; 65 °C, 5 s; 95 °C, 15 s) was conducted. Standard curves for the *pmoA* gene were created using ten-fold serial dilutions of plasmid DNA from 10^2^ to 10^8^ copies/μL. The qPCR amplification efficiency ranged from 91.8 to 94.5%, with an R^2^ of 0.99-1.

**DNA-SIP fractionation**

The isopycnic centrifugation of the 7-day ^13^C-labeled and unlabeled DNA was performed as previously described [22]. Firstly, 2 μg of purified DNA was mixed with gradient buffer (100 mM Tris-HCl, pH=8.0; 100 mM KCl; 1.0 mM EDTA, pH 8.0) and 1.85 g L^-1^ CsCl solution to get a solution with a buoyant density of 1.725 g mL^-1^ and refractive index of 1.4029±0.0002 (nD-TC), which was measured by an AR200 refractometer (Reichert, USA). Secondly, the gradient solution was sealed in an 8-mL centrifuge tube, and an ultracentrifuge (Beckman Optima MAX-XP) was used to centrifuge these tubes at 180,000 ×g at 20°C for 44 h. After centrifugation, the gradient solution was divided into 15 fractions from bottom to top using a peristaltic pump (Baoding Longer Precision Pump Co., Ltd.) by injecting sterile water from the top of the tube at a constant flow rate of 0.53 mL min^-1^. Each fraction was collected in a new sterile 2 mL tube. The buoyant density of each fraction was converted according to its refractive index. The fractionated DNA solution was mixed with 700 μL PEG6000 solution and incubated at 37 °C for 2 h. The tubes were centrifuged in a benchtop microcentrifuge (Eppendorf 5424R) at 13000 ×g at 20°C for 30 min. After discarding the supernatant, 500 μL of 70% ethanol was added to rinse the DNA, and the mixture was centrifuged at 13000 ×g for 10 min. The ethanol cleaning step was then repeated after discarding the supernatant. The DNA precipitate was air-dried, diluted in 30 μL of elution buffer, and kept at -20°C for subsequent analysis.

**PCR amplification and high-throughput sequencing**

For bacterial 16S rRNA gene, the primers 515F (5’- GTGCCAGCMGCCGCGGTAA) and 907R (5’-CCGYCAATTYMTTTRAGTTT) (Angenent et al., 2005) with sample-specific 12-bp barcodes were used. PCR was performed in 50 µL volumes containing 1 µL of each primer (10 µM), 25 µL of Accurate Taq Master Mix (2×), 1 µL of the template, and 22 µL sterile water. Cycling was performed with an initial denaturation at 94°C for 3 min, followed by 32 cycles: 94°C for 30 s, 55°C for 30 s, 72°C for 30 s, and a final extension at 72°C for 10 min on a MiniAmp Thermal Cycler instrument (Applied Biosystems, Foster City, CA, USA). The PCR products were purified with Omega Bio-Tek E.Z.N.A.® Cycle Pure Kit, and their concentration was determined. The purified PCR products were pooled in an equimolar concentration for the Illumina NovaSeq 6000 sequencing system using PE250 cycle combination mode at Beijing Novogene Bioinformatics Technology Co., Ltd. (Beijing, China).

The primer pair A189F/mb661r with sample-specific 8-bp barcodes was used to perform *pmoA* genes PCR amplification in total DNA from incubated sediment. PCR amplification was performed using Accurate Taq Master Mix (2×) in 50 μL volumes with a MiniAmp Thermal Cycler instrument. Cycling consisted of an initial denaturation at 94°C for 4 min, followed by 38 cycles: 94°C for 1 min, 54°C for 1 min, 72°C for 1 min, with a final extension at 72°C for 10 min. The PCR products were then purified, quantified, and mixed in a pool at an equimolar amount. Sequencing was performed using HiSeq2500 (Illumina) PE300 by Shanghai Biozeron Biotechnology Co., Ltd. (Shanghai, China).

Figure S1. The relative abundance of sedimental bacterial phyla in 3 incubation times (*In-situ*, Day 7, Day 21) in ten lakes.
